# Socioeconomic Differences and the Potential Role of Tribes in Young People’s Food and Drink Purchasing Outside School at Lunchtime

**DOI:** 10.3390/ijerph16142447

**Published:** 2019-07-10

**Authors:** Ariadne Beatrice Kapetanaki, Wendy J. Wills, Giada Danesi, Neil H. Spencer

**Affiliations:** 1 The York Management School, University of York, Heslington, York YO10 5DD, UK; 2 Centre for Research in Public Health and Community Care (CRIPACC), University of Hertfordshire, College Lane, Hatfield, Hertfordshire AL10 9AB, UK; 3 University of Lausanne, Faculty of Social and Political Sciences, UNIL-Géopolis, CH-1015 Lausanne, Switzerland; 4 Department of Marketing and Enterprise, Hertfordshire Business School, University of Hertfordshire, de Havilland Campus, Hatfield, Hertfordshire AL10 9AB, UK

**Keywords:** tribal theory, socioeconomic status, schools, food, marketing communications, peer influence

## Abstract

Socioeconomic deprivation has been linked to food consumption practices, but studies investigating the food environment around schools provide mixed findings. Peer influence and marketing cues are considered important influencers of young people’s behaviors. This study used a tribal theory lens to investigate the factors affecting pupils’ purchasing and consumption of food/drinks outside schools at lunchtime. A survey was conducted with 243 pupils from seven UK secondary schools of differing socioeconomic status (SES). A purchasing recall questionnaire (PRQ) was developed and administered online at the participating schools to capture food and drink purchasing, intake, and expenditure. No significant differences were found in terms of energy and nutrients consumed or food/drink expenditure between pupils from schools of lower and higher SES. Enjoyment of food shopping with friends was linked with higher food energy intake and spend. Higher susceptibility to peer influence was associated with greater influence from food advertising and endorsements. Without ignoring the impact that SES can have on young people’s food choices, we suggest that tribal theory can be additionally used to understand pupils’ eating behaviors and we present implications for social marketers and policy makers.

## 1. Introduction

Obesity and diet-related non communicable diseases are major public health issues that affect children, young people, and adults [1,2]. Investment in and greater understanding about young people’s consumption practices is needed to bring about concurrent and future economic and societal benefits through improved nutritional status and health [3].

School time is an important part of an adolescent’s life as they have to make independent choices while being exposed to peer pressure without the direct influence of their family [4]. One way of exercising this independence is through the food choices made during the school lunch break. Some young people purchase their lunch from retailers outside their school and it has been found that the percentage doing so is considerably higher in Scotland (hence the focus of this study) than in Canada and the USA [5,6]. This is important because food and drink consumed outside of home or school is often higher in fat, sugar, or salt [7,8].

Our study focuses on Scotland because current evidence suggests that the percentage of food energy from added sugars (14.1%) is higher among children and adults than the Scottish Dietary Goal of less than 11% of food energy [9]. In 2015, 28% of children from 2–15 years old were at risk of overweight, including 15% at risk of obesity, and the obesity risk was nearly double for children in socioeconomically deprived areas of Scotland [10]. Therefore, understanding whether socioeconomic status (SES) is a key factor for food consumed during the school day may help to find solutions for improving young people’s dietary health.

For young people, food consumption is influenced by a combination of environmental, individual, and social aspects [11]. Previous studies have linked young people’s less healthy food practices to peer influence (e.g., [11,12,13,14,15,16]). In addition, marketing communication techniques can influence the way young people form a social identity and sense of belonging by influencing their purchasing behaviors [17,18].

Socioeconomic deprivation is also thought to shape food consumption practices, and early life socioeconomic status (SES) has been associated with greater risk of obesity during adulthood [19]. A European study illustrated that SES impacts young people’s diets [20], while two recent USA studies found associations between neighborhood socioeconomic deprivation and obesity [21,22]. One way that socioeconomic deprivation is hypothesized to influence food intake is in relation to the density and type of food retailers located in the vicinity of schools (e.g., [23,24,25,26,27]). A second way is via the social shaping of food practices among young people who share a similar SES (e.g., [28,29]). However, the evidence is not clear-cut and further research is needed to investigate and explain associations between SES and food consumption (e.g., [25,30,31,32,33]). This is supported by wider studies which have tried to link economic inequalities to health outcomes but did not find significant relationships (see, for example, [34,35]), which makes this area in need of further research to better understand these dynamics.

The purpose of this study is to investigate lunchtime food consumption and spend outside school, differences based on SES, and the relationships between school peers, marketing cues, and food practices to further our understanding and inform public policy. To build our hypotheses, analyse and interpret our findings, a tribal theory lens was adopted. This is because, as will be illustrated in the next section, young people’s consumption activities have been linked to their need for socialisation and belonging to a community. Our study builds on previous research to understand teenagers’ food choices (e.g., [13,16,29,36]), and responds to calls for further examining neighborhood SES, along with other contextual and social factors, and the impact on food consumption and obesity (e.g., [15,22,32,37,38]).

## 2. Theoretical Background & Hypotheses

### 2.1. Consumer Tribes as A Temporary Escape

In this paper, we use a tribal theory lens to better understand the food and drink intakes of young people at schools with differing SES and the interactions with peers and marketing communication cues. While interacting with consumable resources to establish emotional and hedonistic social relationships, young people can be said to develop a ‘tribe’ [39]. Goulding, et al. [40] call these groups ‘consumer tribes’ as they are held together by consumption activities, which can be temporary. Belonging to a tribe is a consequence of a wish to share similar experiences [40,41]. It is, therefore, possible for young people to aim to belong to more than one tribe to establish differing identities with different groups.

Formation of tribes has been witnessed in both teenagers and adults [41]. Bennett [42] supports the idea that the best way to study young people is in the context of ‘tribes’, following Maffesoli’s [43] tribal theory, where tribes are the modern way for consumer efforts to establish an identity and reconstruct themselves. Such tribes have also been seen as a ‘temporary escape’; a way of escaping from everyday pressures, and not as groups that dominate people’s lives [44]. In this context, teenagers can easily move from one tribe to another in a process of building their own identity, expressing themselves in a collective way for a brief period of time. This tribalism concept is “less a question of belonging to a gang, a family or a community than of switching from one group to another” [43] and it is linked to the origins of mass consumerism [42]. According to this tribalism concept, for example, food shopping patterns of teenagers can be seen as a temporary ‘lifestyle’, rather a ‘way of life’, which can be chosen regardless of class background, gender, or ethnicity. In that sense, driving from Bennett’s [42] example of people consuming various kinds of music under different circumstances, regardless of their SES, teenagers may consume certain food and drinks when they are with their friends during school time and act differently when they are with other peers, in other settings, or when they are with family members.

The driving factor of the practice is the generation of social links during a lunchtime ‘escape’ from school [45], and this can be achieved using marketplace resources [46]. For example, the concept of tribalism could be interpreted as the co-existence of more or less socioeconomically deprived pupils enjoying a shared experience of eating beyond the school gate during their lunch break and, together, consuming similar food and drinks.

Studies on tribes and tribalism often focus on hedonistic choices, such as music consumption (e.g., [42]), clubbing [47], and branded materials [48], but not on more utilitarian items, such as food and drinks. It is also important to consider that the food available around schools may often be unbranded, in independent takeaways for example [49].

Drawing from the above discussion, it is hypothesized that for pupils who consume food and drinks outside the school gate during the school lunch break:

**H1:**  *There will be no significant difference in food and drink (a) intake and (b) spend between pupils in higher and lower SES schools.*

### 2.2. Enjoyment as a Social Facilitator

Belonging to a tribe is a consequence of a wish to share similar experiences and develop desirable social links [41,44]. The effort to belong in a tribe stems from the enjoyment that tribe members get from those shared experiences, and tribalism works as a way of escape and enjoyment [40]. Young people who actively try to establish their own identity [50,51] and achieve a sense of belonging [17,52] might have a great urge to belong to a tribe.

Previous research has found shopping with friends a positive experience, particularly for young people, since it facilitates social comparisons, accomplishment of social needs, as well as bonding with significant others [53,54]. At the same time, evidence shows that shopping with friends can increase the urge to shop, shopping hedonism, and impulsive purchases [55,56]. Studies have also linked eating with others and the number of people eating together with type and quantity of food consumed, suggesting that people tend to eat more when eating with others due to social facilitation [57,58,59,60,61,62,63]. Enjoying shopping with friends also increases perceived social support, which has been found to reduce any negative effect of spending money [64]. If we combine these arguments with tribal theory, in which enjoyment is paramount, it could be hypothesized that during the school lunch break:

**H2:**  *Those pupils who enjoy shopping with friends are more likely to (a) consume and (b) spend more on food and drinks.*

### 2.3. Tribes in Marketing

Marketers have used the concept of tribes to design optimal marketing campaigns that promote a feeling of belonging and communal gathering [41]. ‘Tribal marketing’ focuses on the fact that individuals may be willing to move outside the traditional demographic characteristics used by marketers to segment and target markets [40]. It is based on promoting a sense of belonging and on the notion that consumers look for experiences (including products and services) that can link them to others in an effort to belong to a certain group of people [41]. Marketing techniques, such as advertising, celebrity/cartoon endorsement, and sponsorships often work as a way to build social identity and enhance a sense of belonging [17]. Advertising aimed at young people and children often uses techniques that are appealing to this target group, such as cartoons, animals, celebrities, and humor [65,66], and it has been found to influence food choice and product awareness [67,68]. Endorsers act as role models who can potentially influence consumption decisions by transferring cultural meaning from the celebrity to the advertised product [69]. Young people are prone to influence from role models due to similar processes triggered during normative peer influence and this facilitates adoption of certain life styles and self-image that the role models endorse [70]. From a tribal theory perspective, it might be expected that, during the school lunch break, young people who are more susceptible to their friend’s normative influence in an effort to develop stronger social links with them will be influenced more by food and drink marketing cues. Therefore, it is hypothesized that during the school lunch break:

**H3:**  *Pupils with higher susceptibility to normative peer influence will be influenced more by (a) advertising and (b) endorsement when choosing food and drinks.*

## 3. Materials and Methods

### 3.1. Setting

A survey was conducted among 13–15 years old pupils from seven schools across Scotland. Permission to contact the schools was received from five local authorities in the North, South, East, and West of the country. The schools differed in terms of their SES, according to the Scottish Index of Multiple Deprivation (SIMD) (Table 1). Each school had a set time for the lunch break, which varied from 40–60 min among the schools studied, and pupils could go outside the school during lunchtime.

Ethics approval from the committee, with designated authority at the University of Hertfordshire (HSK SF/UH/00045), was obtained before fieldwork commenced.

### 3.2. Sample

All the parents of young people aged 13–15 years at the seven selected schools were sent information about the study. All pupils were informed about the research, via year group assemblies, classroom visits, and leaflets. Pupils were informed that by completing the questionnaire they were assenting to participate; personal details such as names were not collected. A total of 535 pupils completed the survey across the seven schools. From those, 287 pupils reported purchasing food and/or drink during their lunch break outside their school, and we were able to calculate food intakes for 243 of these pupils. This paper focuses on this group (see Table 1).

A sample of this size is sufficient to provide power in excess of 90% to detect a standardised effect size of 0.5 (often regarded as medium-sized) when conducting a *t*-test to compare “low” and “high” SES groups, using the 5% level of significance. We can thus be reassured that the analyses reported below have sufficient power to allow reasonable conclusions to be drawn.

### 3.3. Questionnaire

An online food and drink purchasing recall questionnaire (PRQ) was developed. This was adapted from the ‘Food and Drink on School Days’ questionnaire used in a 2010 survey of a representative sample of young people in Scotland (version 10) [24]. The PRQ asked pupils what they ate/drank at lunchtime, what food and drink they purchased during the lunch period, where it was purchased from, information on brand and portion size, how much items cost, and how much of the purchased item they consumed. A second section of the PRQ included scales to gather information about friends/peers and marketing influences when shopping for food and drinks during lunch break outside the school.

A pilot study was conducted, testing the wording and pupils’ ability to complete the questionnaire, to test the duration for completion and check for technical problems with the online version.

The survey was completed online via PCs and laptops in the classroom and, when possible, immediately following the lunch period to minimize misreporting [71]. In some cases, it was impossible for schools to organise for the PRQ to be completed after lunch, often because PCs or laptops were not available. In such cases, the PRQ was administered in the morning and pupils were instructed to provide information about lunchtime purchases from the previous day.

### 3.4. Variables

#### 3.4.1. Food and Drink Intake

Through the PRQ, we captured information on food and drink purchasing, brand, product size, the retailer where the purchase was made from, and the amount consumed. With this information, we identified the products consumed by pupils and calculated the energy intake (KJ), salt, sugar, fat, and saturated fat intake via the information included on the packaging or from online information, for branded products. For unbranded items, such as a sandwich bought from a bakery or chips/fries, the research team purchased the product and weighed its different components using digital scales; nutrient information was then calculated from McCance and Widdowson food composition tables using Diet Plan software (version 6.0, Forestfield Software Ltd, Horsham, UK)). For example, for a filled roll, the item was purchased and the individual components were weighed and entered into the software separately (e.g., cheese, cucumber). For components that were unable to be checked for portion size (e.g., ketchup, butter), an assumed weight was used, based on the suggested weight available in Diet Plan.

In addition, the research team calculated whether intake was above or below the Nutrient Standards for School Lunches (NSS) for pupils in secondary schools, which states that lunch should contribute no more than a third of a young person’s daily nutritional needs for a healthy diet [72].

#### 3.4.2. Expenditure

We asked pupils to provide the cost of the product they reported consuming during the lunch break. When there was no cost provided, the researchers used the information available to identify the product and track down its price by visiting the retailer.

#### 3.4.3. Susceptibility to Peer Influence

Susceptibility to normative peer influence was measured using two items from the Bearden, et al. [73] scale, including ‘I buy the food/drink that my friends will approve of ’ and ‘I feel part of my group of friends when I purchase the same food/drinks that they purchase’. All responses were recorded on a seven-point scale, ranging from “Strongly disagree” (scoring 1) to “Strongly agree” (scoring 7).

#### 3.4.4. Shopping Enjoyment

Two items taken from Mangleburg, Doney, and Bristol [53] were used to measure shopping enjoyment with friends, including ‘I like to shop with friends for food/drink during the lunch break’ and ‘it is fun to shop with friends for food/drink during the lunch break’. All responses were recorded on a seven-point scale ranging from “Strongly disagree” (scoring 1) to “Strongly agree” (scoring 7).

#### 3.4.5. Advertising and Endorsement Influence Scales

Two scales on marketing communication elements were developed to measure advertising influence and endorsement influence. A seven-point scale ranging from “Not important at all” (scoring 1) to “Very important” (scoring 7) were used to capture pupils’ responses on these items. Advertising influence included three items on TV, online, and other advertisements. Endorsement influence was constructed from three items on celebrities and cartoon endorsement and sponsorships.

Each of the above scales were created by summation of the answers to each question. The scales have Cronbach’s alpha values in excess of the 0.7 benchmark (Table 2), commonly used to indicate an effective scale [74].

### 3.5. Data Analysis

The seven schools were categorized as high SES or low SES, basedon the neighborhood in which they were located, using SIMD deciles. A dichotomous categorization was important to have sufficient numbers within each group for the analysis and was also in line with the relevant literature. Four schools were categorized on this basis as low SES (*n* = 149 pupils) and three were categorized as high SES (*n* = 94 pupils) (Table 1). Data were analyzed using the software package IBM SPSS version 23 (IBM Corp., New York, NY, USA).

To test the hypotheses, we took account of two issues: (i) The distribution of the variables and (ii) clustering.

The variables being analyzed were frequently non-normal in their raw form, so attempts were made to transform the non-normal distributions. For analyses that compare pupils attending ‘low SES’ schools with pupils from schools in the ‘high SES’ category, we determined whether or not it was appropriate to undertake a two-sample *t*-test, looking at sample sizes in each group, sample variances, and skewness [75].

The clustering of cases must also be addressed because it may be the case that pupils in the same school are more similar to pupils across different schools due to neighborhood, school, or other effects. The extent of this clustering can be measured by the intra-class correlation coefficient. If this clustering is non-trivial then traditional analysis methods will over-estimate the accuracy with which estimates can be made. To address this issue, we used multilevel modelling strategies which explicitly allow for the clustering in the data.

When multilevel modelling was not possible, Spearman’s rank correlation was used to assess the strength of the relationship between scales. This takes account of the lack of normality, but the fact that the data are clustered (pupils within schools) means that traditional routes to assessing the statistical significance of these correlations are less appropriate than we would like them to be. Confidence intervals were used for assessing both the size of the correlation and the uncertainty associated with it. To overcome the clustering issues, we have opted for bootstrap confidence intervals, where repeated samples of the data are taken and, from these, a confidence interval is created. In this study, there were only seven schools tested, and, as a result, the traditional resampling of individuals rather than clusters must be undertaken. However, awareness of the possible impact of the clustering is ensured when discussing the findings and drawing conclusions.

When discussing the findings below, it is acknowledged that lack of evidence for an association is not the same as evidence of lack of an association. However, given the sample size, there is a good degree of statistical power. It is, therefore, reasonable to conclude that if associations did exist, there is a good chance of observing them.

## 4. Results

### 4.1. Descriptive Statistics

Table 3 shows the reported mean consumption of and expenditure on food and drinks and the percentage of pupils who exceeded NSS guidelines [72].

### 4.2. Hypothesis Testing

For all five variables (KJ, fat, saturated fat, sugar, and salt), there were no statistically significant differences between the socioeconomic groups. We can say this where multilevel modelling was possible (for energy and sugar consumption, because formal hypothesis tests were conducted (resulting in p-values of 0.808 for energy and 0.551 for sugar). For the other three variables, it was not possible to conduct a formal hypothesis tests using multilevel modelling. However, as can be seen in Table 4, for each variable there were large overlaps in confidence intervals for the socioeconomic groups. We can thus say with assurance that no statistically significant differences exist.

We also examined if there was an association between consuming KJ, fat, saturated fat, salt, or sugar at levels above NSS recommendations and the two categories of SES. Using multilevel modelling for binary outcomes, we found that, for all five variables, there was insufficient evidence to claim that an association existed (for KJ, *p* = 0.615; for fat, *p* = 0.288; for saturated fat, *p* = 0.594; for salt, *p* = 0.244; for sugar, *p* = 0.793). Therefore, we accepted the hypothesis (H1a) that there are no statistically significant differences of energy (KJ) and nutrient (fat, saturated fat, salt, sugar) intake between pupils in areas with higher or lower SES.

Multilevel modelling of the (log transformed) cost indicated that there is insufficient evidence to claim the existence of differences between ‘high’ and ‘low’ SES schools in terms of the spend on food and drink amongst those who get food outside school at lunchtime (M_lowSES_ = £2.15, M_highSES_ = £2.17, *p* = 0.768). Therefore, we accept the hypothesis (H1b) that there will be no statistically significant differences of food and drink expenditure between pupils in areas of high and low SES.

Spearman’s rank correlation was used to examine the relationship between shopping enjoyment and consumption levels/cost. Table 5 shows marginal evidence for a positive relationship for energy, fat, and spending (the latter meaning H2b is accepted), but insufficient evidence for saturated fat, salt, or sugar. Therefore, there is evidence to accept the hypothesis (H2a) that those who enjoy shopping with friends consume more than those who do not enjoy shopping with friends.

Finally, Spearman’s rank correlation was used to examine the relationship between shopping enjoyment and marketing techniques. As shown in Table 6, there is sufficient evidence to claim that those with higher susceptibility to normative peer influence are influenced more by advertising and endorsements. For advertising, the relationship is stronger in ‘low’ SES cases than in ‘high’ SES cases. Therefore, H3a and H3b are accepted.

## 5. Discussion

In this study, we found no significant difference in terms of energy and nutrients consumed and food/drink expenditure during the lunch break between young people from schools of lower and higher SES. Therefore, tribal theory might be a helpful framework to study the dietary patterns of young people who buy and consume lunch outside school at lunchtime [46]. It is important to highlight that SES may have a different meaning in different populations and that socioeconomic deprivation can be more or less severe among different communities and households, so for those pupils without the financial ability to purchase food outside their school the tribal approach may be irrelevant. However, the focus of our study is on those pupils from different socioeconomic backgrounds who have the financial ability to purchase food and drinks during their lunchbreak, and, for those, an interpretation using the tribal concept might be helpful in understanding their consumption practices.

Tribal theory might also explain why, while studies indicate that obesity rates, for example, differ between areas of higher and lower SES (e.g., [9,38,76,77]), our findings suggest that there are other influencing factors for young people when they are with their peers during the school day. It might be, for example, that young people from lower SES backgrounds eat and shop according to social tribes during the school lunch break, whereas their food and drink consumption when at home might be more associated with the familial SES (influencing how much money parents spend on food and from which retailers they shop from). Tribal theory might also explain why studies investigating the food environment around schools provide inconclusive and mixed findings (e.g., [23,78,79,80]). The school lunch break offers a period for escaping the stress or pressure of school and a time to enhance social links and identity, with SES being a less important factor for what food and drink is consumed than at other time points [44,45].

That is not to discount SES as an important structural and social factor overall, but in order to address inequalities in health it is useful to consider additional factors influencing young people, such as the need to enjoy their school day by socializing via food and drink consumption practices. Shildrick and MacDonald [81] criticize post-subcultural studies for being biased and failing to capture the importance of class background and other socioeconomic attributes when studying youth behavior and culture. Others [82,83,84] have shown that “contemporary youth culture remains deeply divided” [81] due to social inequalities and SES should still be taken into account when young people are studied. This study adopts the concept of tribes to explain a specific behavior sensitive to peer interactions; however, the concept of tribes cannot totally replace the idea that SES is important for other behaviors and contexts. Therefore, tribal theory could be used as an additional, rather than an alternative, explanation of certain practices, and future studies are needed around tribalism and food consumption to further investigate and validate these arguments.

Overall, and regardless of SES, we found higher optimal energy, fat, salt, and sugar intake among young people consuming food and drink outside school, in relation to the NSS guidelines (Table 3). This is in line with previous studies, which found that less healthy food options are popular among young people [13,29], and this can also be fueled by the highly obesogenic environment witnessed around schools regardless of SES [85,86]. In addition to this, our findings showed that more pupils from lower SES than higher SES schools chose to purchase food outside of their school and, therefore, are potentially more exposed to unhealthier food options, since the food provided by the school canteens meets the NSS guidelines. Current calls for action focus on more deprived areas, but our findings are crucial in realizing that not only pupils from low SES, but also pupils from high SES schools are susceptible to less healthy food and drink options during the school lunch break, hence universal as well as targeted policies and actions are needed. The study of Black, et al. [33], who found that differences in SES did not affect food availability or the cheapest price provided by retailers, also supports the above arguments. However, this does not imply that social inequalities can be ignored. Factors underpinning food purchasing and eating practices, such as being in a pleasant and welcoming school environment [87] where socialization is encouraged, are likely to contribute to higher numbers of pupils consuming healthy food at school. This minimizes additional factors related to the inequalities observed in the wider environment of young people (e.g., due to the family eating environment or the environment offered by commercial retail outlets).

Enjoying shopping with friends was found to be related to higher expenditure and higher energy intake in line with previous studies that found links between shopping with friends and purchasing practices (e.g., [88,89]). Using social contagion theory [90], these findings might be explained partly because young people want to be accepted by significant others through consuming certain things (e.g., [16,91,92,93]). From a social facilitation perspective [94], these findings might be explained as a form of impulsive food purchase and consumption because young people, when they perform food practices with friends, are distracted by enjoying their friends’ company [55,62,95].

Our findings show that eating with friends to socialize is very important for young people and SES was found to have no significant impact on their food intake and spend, and their food and drink intake, measured in the form of energy, salt, sugar, and fat, during the lunch break was high. If pupils want to be eating with friends to socialize, but do not have much money to spend, a marketing environment around schools that offers low prices on foods high in fat, sugar. or salt, through sales promotions, meal deals, price discounts, and celebrity/cartoon endorsements [12,96] facilitates the consumption of less healthy foodstuffs [23,24]. Environmental changes are, therefore, vital to support tribes in consuming healthier food and drinks (within or outside school) without compromising their desire for enjoyment and socialization during the lunch break.

Finally, we found that those with higher susceptibility to normative peer influence will be prone to marketing techniques, such as advertising, endorsements, and sponsorships. This might explain why young people are often susceptible to marketing practices and end up consuming less healthy food and drinks [18,67,68].

## 6. Conclusions and Policy Implications

Our findings suggest that pupils who consume food and drinks during their lunch break are influenced by their enjoyment of shopping with friends. This is in line with the tribal theory concept that sees people coming together regardless of their demographic background to experience a specific ‘lifestyle’, and food consumption is part of this. Therefore, our study contributes to the ongoing debate on post-subculture theory [81,97] and supports that tribal theory could provide an additional perspective for exploring youth food consumption along with any influences of the food environment and SES, which have led previous studies to produce ambiguous findings.

The food environment around schools differs to other food environments, as young people are not directly influenced by their family members at this time of the day. Marketing cues and peers affect their eating behaviors and our study shows that a worrying percentage of pupils who purchase food beyond the school gate exceed the NSS guidelines for energy, sugar, salt, and fat intake. A holistic approach is required, which takes into account whether regulation of food retailing in the vicinity of schools is feasible and likely to be effective. Young people can access the food environment on the way to, during, and after school; therefore, regulations on what the commercial sector can sell around school in isolation is not likely to solve the problem. As a result, schools have an important role to play in shaping pupils’ food decisions. Enhancements within school dining spaces are certainly needed, drawing on this study’s findings about young people’s need to socialize and be part of a social tribe. Schools can be safe places that protect pupils from other food-related environmental influences, such as possible less healthy-eating family practices and/or unhealthy food environments in the wider neighborhood, which may be more pertinent in lower SES areas. Social marketing techniques could be useful to help achieve this by using tribal marketing approaches [41,48] to promote the school dining environment. This would promote healthy eating as a way to link with others, to be part of a school peer tribe, and to therefore have an enjoyable school lunch break (escapism).

Our marketing-related findings can also be used by policy makers to enforce unhealthy food marketing regulations to better protect young people who are prone to peer influence and materialism and have a strong need to belong and shape their identities through food consumption practices. These regulations, to be effective, should draw on celebrity endorsements and sponsorships, on mass media communications, including social media, and on branding strategies. Since our findings found similar food and drink intake in both deprived and less deprived areas, these initiatives should be targeting areas of both high and low SES.

## 7. Limitations and Future Research

The study has limitations that must be considered. The collection of data from pupils in schools relies on them correctly recalling what they bought/consumed at lunchtime. In some cases, pupils had to recall what they had consumed the previous day, which may have caused issues of misreporting; however, the fact that, in most cases, the data were collected soon after the lunch break will have minimized issues caused by this. Furthermore, the number of schools included in the study was quite limited. Future studies should consider a bigger sample of schools or young people to derive comparisons among areas of high and low SES. Finally, this study focuses on pupils’ food and drink intake during the school lunch break, so other aspects, such as general food habits, dietary patterns, and food groups intake, could be considered in future studies. Therefore, a broader study to understand the daily food consumption of pupils (throughout the school day, rather than just at lunchtime) and how friends, family, and marketing aspects influence their choices and overall food and drink intake could shed more light on this important area and better inform future policies and industry practices in order to improve the diet and health of young people.

## Figures and Tables

**Table 1:** Participating schools in terms of the Scottish Index of Multiple Deprivation (SIMD) category.

School ID	SIMD Decile ^a^	SES Classification	Girls	Boys	TOTAL
Sch1	1	Low	26	24	50
Sch2	1	Low	10	22	32
Sch3	1	Low	14	13	27
Sch4	3	High	30	24	54
Sch5	1	Low	24	16	40
Sch6	3	High	1	11	12
Sch7	2	High	18	10	28
			123	120	243

^a^ 1 = SIMD ranks 1–2602 (four most deprived deciles); 2 = SIMD ranks 2603–3903 (two middle deciles); 3 = SIMD ranks 3904–6505 (four least deprived deciles). For details see: http://www.gov.scot/Topics/Statistics/SIMD.

**Table 2:** Peer influence and marketing constructs.

Construct	Number of Items in Scale	Cronbach’s Alpha	*N*	Mean	Standard Deviation
Susceptibility to normative peer pressure	2	0.86	211	5.58	3.50
Shopping enjoyment	2	0.79	211	10.02	3.38
Advertising influence	3	0.95	211	8.18	5.32
Endorsement influence	3	0.89	211	8.37	5.32

**Table 3:** Food/drinks intake and expenditure during lunch break outside the school gate on the day of the survey (*N* = 243) compared to the Nutrient Standards for School Lunches (NSS) values.

	Mean (Bootstrap 95% Confidence Interval *)	Max Value from Lunch Intake Based on NSS ** [72]	% of Pupils Above NSS
Energy (KJ)	2019.79 (1827.26, 2205.39)	2776 ***	28%
Fat (g)	19.57 (16.85, 22.23)	25.8	32%
Saturated fat (g)	6.48 (5.39, 7.51)	8.1	29%
Salt (g)	2.05 (1.30, 2.62)	2.06	31%
Sugar (g)	31.83 (28.54, 35.08)	19.5	64%
Expenditure (£)	2.16 (1.95, 2.36)	n/a	n/a

* Bootstrap 95% confidence intervals using 5000 replications. ** Nutrient Standards for School Lunches (NSS) for pupils in secondary schools [72]. *** According to the NSS [72], the amount of energy shall be either 2776 or within 10% of this.

**Table 4:** Means and bootstrap confidence intervals for nutritional variables by socioeconomic status (SES).

	Mean and Bootstrap 95% Confidence Interval * for “low” SES	Mean and Bootstrap 95% Confidence Interval * for “high” SES
Energy (KJ)	2032.75 (1764.56, 2301.76)	1999.24 (1740.92, 2256.85)
Fat (g)	20.30 (16.31, 24.04)	18.42 (14.95, 21.67)
Saturated fat (g)	7.30 (5.72, 8.80)	5.17 (3.80, 6.37)
Salt (g)	1.77 (0.98, 2.34)	2.48 (1.06, 3.59)
Sugar (g)	31.56 (27.31, 35.43)	32.27 (26.50, 37.63)

* Bootstrap 95% confidence intervals using 5000 replications.

**Table 5:** Correlations between enjoying shopping with friends, food/drinks intake, and expenditure.

	Spearman’s Correlation and Bootstrap 95% Confidence Interval *
Energy	0.18 (0.05, 0.31)
Fat	0.19 (0.06, 0.33)
Saturated fat	0.15 (0.01, 0.28)
Salt	0.13 (0.01, 0.26)
Sugar	0.01 (−0.12, 0.15)
Expenditure	0.18 (0.05, 0.31)

* Bootstrap 95% confidence intervals using 5000 replications.

**Table 6:** Correlations between normative peer influence and marketing techniques.

	Marketing Technique	Spearman’s Correlation and Bootstrap 95% Confidence Interval *
**Normative Peer Influence**	Advertising	0.40 (0.28, 0.54)
	Endorsement	0.43 (0.31, 0.56)

* Bootstrap 95% confidence intervals using 5000 replications.
